# SEMbeddings: how to evaluate model misfit before data collection using large-language models

**DOI:** 10.3389/fpsyg.2024.1433339

**Published:** 2025-02-04

**Authors:** Tommaso Feraco, Enrico Toffalini

**Affiliations:** Department of General Psychology, University of Padova, Padua, Italy

**Keywords:** large language models, artificial intelligence, confirmatory factor analysis, validity, assessment, structural equation models, modification indices

## Abstract

**Introduction:**

Recent developments suggest that Large Language Models (LLMs) provide a promising approach for approximating empirical correlation matrices of item responses by utilizing item embeddings and their cosine similarities. In this paper, we introduce a novel tool, which we label *SEMbeddings*.

**Methods:**

This tool integrates *mpnet-personality* (a fine-tuned embedding model) with latent measurement models to assess model fit or misfit prior to data collection. To support our statement, we apply SEMbeddings to the 96 items of the VIA-IS-P, which measures 24 different character strengths, using responses from 31,697 participants.

**Results:**

Our analysis shows a significant, though not perfect, correlation (*r* = 0.67) between the cosine similarities of embeddings and empirical correlations among items. We then demonstrate how to fit confirmatory factor analyses on the cosine similarity matrices produced by *mpnet-personality* and interpret the outcomes using modification indices. We found that relying on traditional fit indices when using SEMbeddings can be misleading as they often lead to more conservative conclusions compared to empirical results. Nevertheless, they provide valuable suggestions about possible misfit, and we argue that the modification indices obtained from these models could serve as a useful screening tool to make informed decisions about items prior to data collection.

**Discussion:**

As LLMs become increasingly precise and new fine-tuned models are released, these procedures have the potential to deliver more reliable results, potentially transforming the way new questionnaires are developed.

## 1 Introduction

The validity and reliability of measurement tools are fundamental in quantitative sciences. However, in the social sciences, researchers often aim to measure constructs that are not directly observable (Flake and Fried, 2020), relying on statistical techniques to establish the validity of scales. Recent advancements in psychometrics have particularly emphasized latent variables and structural equation modeling (SEM) as the preferred methods for assessing the validity of reflective variables (Markus and Borsboom, 2013). These variables, though not directly observable, are hypothesized to influence individuals’ behaviors and, consequently, their test scores or item endorsements, which are observable. For instance, a person with high levels of state anxiety is likely to endorse items such as “I feel anxious at the moment” and “My hands are sweating,” but not “I like chocolate.” Anxiety, which is unrelated to a preference for chocolate, only influences the correlation between the first two items because they are both explained by the same unobserved variable, whereas liking chocolate is not.

SEM is then used to identify factors that account for the covariation between item scores. Specifically, confirmatory factor analysis (CFA) is employed to detect specific patterns of covariation, i.e., to identify the predetermined factors hypothesized to underlie such covariation. In this process, after researchers have developed a set of items during the substantive phase of construct validity (e.g., through literature review, construct conceptualization, expert item review, item mapping, focus groups, cognitive interviews, etc.) (Flake et al., 2017), data from large and representative samples are collected and analyzed (structural phase of construct validity). CFAs are then used to fit the hypothesized model to the data, and fit indices are inspected to assess model fit or misfit (Flake et al., 2017; Beaujean, 2014; Schermelleh-engel et al., 2003). If the model does not fit the data, researchers conclude that there is likely an issue with the hypothesized model (e.g., the construct may have a different structure or may not influence the items as expected) or with some of the items (e.g., they may have correlated residuals within or between subscales).

When faced with this situation, we need to adjust our model or questionnaire by either dropping items, reformulating them, or writing new ones. Modification indices are commonly used to identify which items are particularly problematic (Hoyle, 2023). Importantly, once the problematic items are identified, a new data collection is necessary to avoid overfitting. If we modify the model based on the current data, it will eventually fit that specific data perfectly. However, conducting a new data collection and analysis is time-consuming and resource-intensive, potentially taking months and requiring significant financial investment, with no guarantee of success. This raises the question: is there a way to reduce this risk?

We propose that large language models (LLMs) can partially address this issue, and we propose a new tool to estimate and detect model misfit without (before) data collection.

### 1.1 Large language models

Recent advancements in artificial intelligence have led to the development of efficient transformer-based large language models (LLMs) that excel in text processing and semantic understanding. These models are not only capable of generating and “understanding” text but are also highly effective at extracting valuable linguistic information.

Three main distinctions can guide the adoption of one model over others: model size, architecture type, and training stage (Hussain et al., 2024). Larger models often (though not always) outperform smaller models, but they require significantly more computational capacity and their performance on specific tasks may be influenced by their architecture type and training stage. Encoder architectures, such as BERT, are optimized for producing accurate embeddings and are particularly useful for feature extraction tasks. Decoder architectures, like GPT, are designed to generate text and are particularly suited for tasks requiring the prediction of tokens in sequence. Encoder-decoder architectures, such as BART, combine the strengths of both encoder and decoder approaches, making them ideal for tasks that require both text comprehension and generation.

Finally, foundation or pretrained models can be fine-tuned on specific datasets to boost performance on specialized tasks. Open-source models are especially appealing because they provide access to model weights, codebases, training procedures, and data sources, facilitating the development of customized, fine-tuned models (Burton et al., 2024).

#### 1.1.1 Large language models and item similarity

The link between LLM and psychological questionnaires is rooted in semantics. Indeed, the use of questionnaires to measure the variability of psychological traits can be traced back to the lexical hypothesis (Goldberg, 1993), which laid the foundation for the widely recognized Big Five model of personality and its associated questionnaires, as well as newer emerging theories like the HEXACO model (Ashton and Lee, 2020). The lexical hypothesis suggests that the most common and significant human behaviors, emotions, and thoughts are encoded into language through words that describe them. For example, some individuals may be described as anxious, nervous, and emotionally unstable, while others may be characterized as calm, stable, and optimistic. These adjectives reflect typical tendencies, commonly referred to as personality traits (in this case, neuroticism). In essence, words exhibit similarities and co-occurrences that partially mirror the underlying behaviors, thoughts, and emotions they represent. Although LLMs and the lexical hypothesis cannot directly detect non-semantic factors that influence item covariation, including social, environmental, contextual, and genetic influences, such semantic similarities and co-occurrences can be effectively captured by modern LLMs (Hussain et al., 2024; Hommel and Arslan, 2024; Wulff and Mata, 2023; Binz and Schulz, 2024; Hommel et al., 2022; Kjell et al., 2023).

Specifically, LLMs represent textual information as vectors of length *n*, where *n* corresponds to the number of hidden properties extracted by the encoder block. These vectors, known as embeddings, map the text into an *n*-dimensional space, allowing the model to effectively capture and represent semantic relationships and contextual information (Hussain et al., 2024). The similarity between two embedding vectors can be calculated using various indices, including cosine similarity (Hussain et al., 2024; Wulff and Mata, 2023; Guenole et al., 2024) that we here adopt in line with previous studies. This yields a single similarity value for each pair of items (e.g., the similarity between the embedding for “I feel anxious at the moment” and “My hands are sweating”). Similar to correlations, these values range from 0 (completely orthogonal vectors) to 1 (overlapping vectors). For example, we would expect a relatively high cosine similarity between the two anxiety-related items mentioned above, whereas the similarity with the item “I like chocolate” would be lower—mirroring the correlations we would observe if these items were administered to hundreds of people.

Recent studies have provided empirical support for this hypothesis, showing that embeddings can effectively address jingle-jangle fallacies between psychological items and scale definitions (Wulff and Mata, 2023). Additionally, embeddings have been used to predict empirical item correlations and fit pseudo factor analyses (Guenole et al., 2024) and network analysis (Russell-Lasalandra et al., 2024). In essence, these findings suggest that it is possible to predict how individuals will respond to specific items and estimate the correlation matrix between items or scales. This information can be used for preliminary assessments of scales’ validity and reliability, allowing researchers to perform *a priori* checks before collecting data (Hommel et al., 2022; Kjell et al., 2023; Russell-Lasalandra et al., 2024).

Based on the distinctions among models outlined above, we adopted the *mpnet-personality* model^1^ to estimate item correlations through embeddings and cosine similarities. The *mpnet-personality* model was fine-tuned on the MPNet architecture, using 200,000 pairs of personality items, and builds on a BERT-based architecture with a decoder structure. This model offers the advantage of being specifically tailored for predicting item correlations, and it outperforms larger, more computationally demanding models in this particular task (Wulff and Mata, 2023).

While the ability of LLMs to predict item and scale correlations has already been established, although to a limited level, here we take it a step further by proposing a method to evaluate model misfit before data collection. This approach combines confirmatory factor analysis, fit indices, and modification indices with researcher judgment and interpretation, providing a robust framework to assess model fit.

### 1.2 Rationale of the study

SEMs and CFAs are commonly used to test the structural validity of questionnaires in psychology. These methods work directly with the covariance or correlation matrix of multiple indicators, such as questionnaire items. Essentially, if we have the correlation matrix for a set of indicators, we can fit any CFA model to this matrix and obtain results equivalent to those that would be derived from data collection used to estimate the same correlation matrix.

Given that LLMs can predict correlations between items, we propose utilizing the matrix of cosine similarities between the items’ embeddings provided by the *mpnet-personality* model to conduct CFAs before data collection, thereby allowing for the *a priori* inspection of model fit or misfit. We termed this procedure “*SEMbedding*” to emphasize the use measurement models on cosine similarities of embedded items. To validate this approach, we utilized the Values in Action Inventory of Strengths-P (VIA-IS-P), a well-established questionnaire designed to measure 24 different character strengths (McGrath, 2019) (see Table 1 for a description of the 24 strengths). The selection of the VIA-IS-P offers distinct advantages for our objective compared to using the personality questionnaires previously examined in similar studies. While the Big Five scales are renowned for their high psychometric validity, the VIA-IS questionnaires have been subject to criticism in the literature, and their validity remains under scrutiny. Furthermore, only a limited number of studies have employed a CFA approach to investigate the validity of the VIA-IS questionnaire, rendering it an ideal candidate for detecting model misfit (Feraco et al., 2022). LLMs in this context allows for the identification of potential issues that may be present within this measure.

**Table 1:** The 24 character strengths.

Character strengths	Components
Appreciation of beauty	Awe, wonder
Bravery	Valor, assertiveness
Creativity	Originality, ingenuity
Curiosity	Interest, novelty seeking, openness to experience
Fairness	Equity, impartiality
Forgiveness	Mercy
Gratitude	Thankfulness
Honesty	Authenticity, integrity
Hope	Optimism, future-mindedness, future orientation
Humility	Modesty
Humor	Playfulness
Judgment	Open-mindedness, critical thinking
Kindness	Generosity, nurturance, care, compassion
Leadership	Guidance, supervision
Love	Closeness, intimacy
Love of learning	Systematically adding knowledge
Perseverance	Persistence, industriousness
Perspective	Wisdom
Prudence	Cautiousness
Self-regulation	Self-control
Social intelligence	Emotional intelligence
Spirituality	Religiousness, faith, purpose
Teamwork	Citizenship, social responsibility, loyalty
Zest	Vitality, enthusiasm, vigor, energy

We thank Dr. Nicole Casali for granting permission to use the table.

Specifically, we:

1. Extracted the embedding vectors for all the items of the scales using the fine-tuned *mpnet-personality* model, which outperforms other available models for our aims (Wulff and Mata, 2023).
2. Calculated the cosine similarity between the embeddings of the items in the VIA-IS-P, which, in the case of the *mpnet-personality* model, directly yield correlation values.
3. Calculated the correlation between the matrix of cosine similarities and true item correlations of the VIA-IS-P items calculated on a large sample of 31,697 participants.
4. Fit CFAs on the cosine similarities matrix and test for model fit or misfit.
5. Compared the results of the CFAs fit on the cosine similarities and those fit on empirical correlation matrices.
6. Used modification indices to interpret why some models do not fit the data well and individuate those items responsible for model misfit.

## 2 Method

### 2.1 Participants

Anonymized data from 31,697 international respondents were gently provided by the VIA Institute on Character. These participants completed the VIA-IS-P directly on the Institute’s website in English and agreed sharing their responses for research purposes.

### 2.2 Materials

The *Values in Action Inventory of Strengths-P* (VIA-IS-P) (McGrath, 2019) is a 96-item questionnaire for measuring character strengths. Each strength is measured with four items scored on a 5-point Likert scale (1 = “Very much unlike me” to 5 = “Very much like me”). The original measure showed high internal consistency for every strength (Cronbach’s alpha range: 0.65–0.87, McGrath, 2019). However, its factorial structure and unidimensionality of the single subscales is not well established (Feraco et al., 2022) and a new analysis might suggest room for change. Embeddings of the 96 items were calculated using the *mpnet-personality* model, but different models could be used (see the open materials available on OSF for a demonstration using the OpenAI embedding model *ada-002*).

### 2.3 Computational analysis: from embeddings to cosine similarities

**Figure 1:**
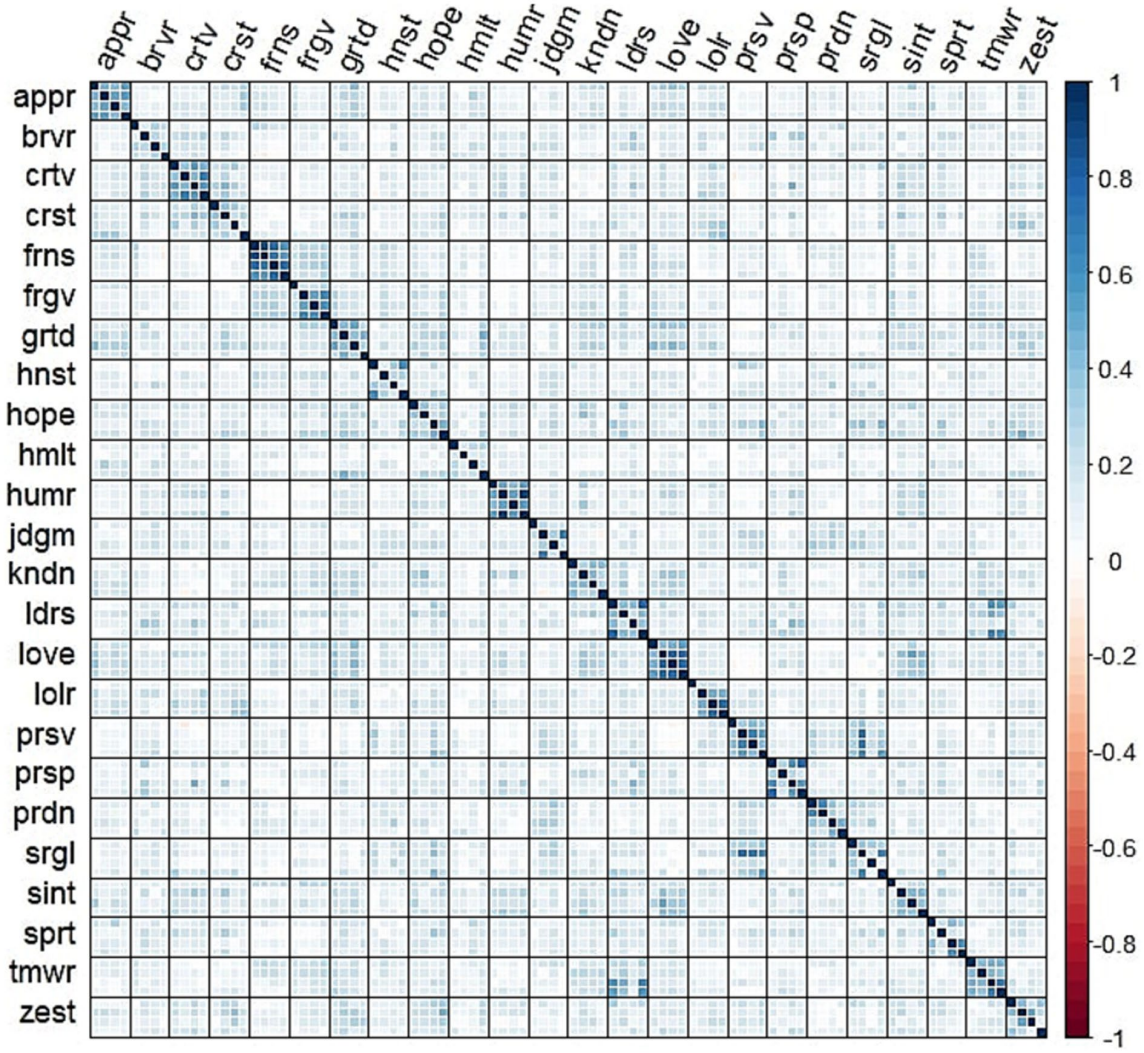
Cosine similarity matrix between the items of the VIA-IS-P. Items are grouped by strength. Each strength is measured by 4 items.

Using the *mpnet-personality* model, we computed the embedding vectors for each of the 96 items of the VIA-IS-P from their respective texts. We calculated the cosine similarity between each embedding vector, resulting in a 96×96 matrix of cosine similarities that can be directly interpreted as a correlation matrix with values of 1 along the diagonal (Wulff and Mata, 2023). The cosine similarities between the items are displayed in Figure 1. Figure 1 shows that each group of four items generally exhibits higher similarity compared to items from other strengths, evident from the 4×4 squares along the diagonal. However, we can already detect some inconsistencies, with certain items within strengths showing low similarity to each other (e.g., humility items), or displaying greater similarity to items from different strengths. For instance, items related to self-regulation exhibited high similarity with items from the perseverance scale. Similarly, items from the teamwork scale demonstrated high similarity with those from the leadership scale. These findings may suggest the need for revising some items or scales to avoid cross-loadings or jingle-jangle fallacies.

## 3 Results

Python 3.11.5 was used to calculate items’ embeddings and their cosine similarity using *mpnet-personality*. R 4.3.1 was used for subsequent analysis, including CFAs, correlations, and plots.

### 3.1 Cosine similarities and empirical correlations

**Figure 2:**
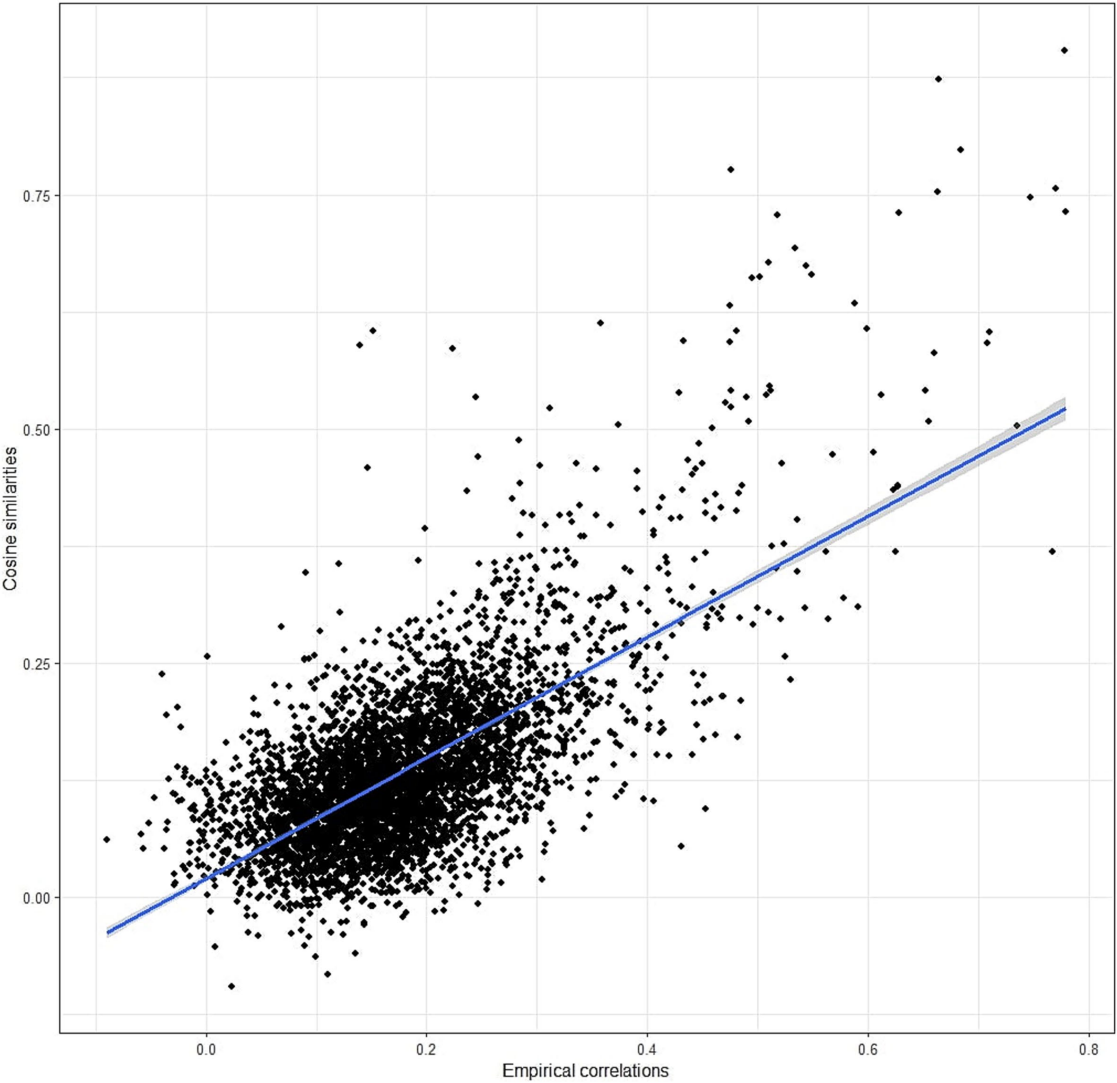
Correlation between empirical correlations and cosine similarities.

While the cosine similarity matrix appears to effectively capture the covariance between items, it may not precisely mirror the empirical correlations between the items. Therefore, we computed the correlations between all items using the collected data and compared them with the cosine similarity matrix. Specifically, we calculated the correlation between the lower triangles of the two matrices (excluding the diagonal). This analysis revealed a correlation of 0.67 (as depicted in Figure 2), indicating that cosine similarities can indeed predict items’ correlations to a significant extent, although a large part of variance remains unexplained.

### 3.2 SEMbeddings: CFAs with cosine similarities matrices

After confirming the comparability of cosine similarities and empirical item correlations, we proceeded to employ CFAs to examine the factorial structure of items prior to data collection. As said before, we label this procedure “SEMbedding.”

In all models, we set *N* to 10,000 to ensure the reliability of fit indices. We evaluated model fit using the comparative fit index (CFI), the Tucker-Lewis index (TLI), the standardized root mean squared residual (SRMR), and the root mean squared error of approximation (RMSEA). We adopted non-stringent cutoffs: CFI and TLI < 0.90 and SRMR and RMSEA >0.08 are considered poor, consistent with previous studies on character strengths (Ng et al., 2017). If a model exhibits two or more poor fit indices, we descriptively conclude that it does not adequately fit the data.

#### 3.2.1 Detecting wrong models with multiple factors

To initially assess the validity of SEMbeddings, we tested the SEMbedding procedure on wrongly specified models to test if this procedure correctly identifies model misfit. To do this, we fit 276 CFAs (all possible combinations of two pairs of strengths). Each CFA involved a single latent variable loading onto items corresponding to two strengths, consistently fitting a unidimensional model when a two-factor model would have been the appropriate choice. The SEMbeddings fit indices effectively detected model misfit in all cases, except in 5% of instances where the SRMR value was lower than 0.08. The fit index values fell within the following ranges: CFI = [0.22; 0.90], TLI = [−0.09; 0.86], SRMR = [0.06; 0.29], RMSEA = [0.09; 0.39]. Results from the empirical data were similar, as we always detected model misfit, except for two CFI values, one TLI value, and one RMSEA value. Again, the SRMR was lower than 0.08 in 4% of the cases.

**Figure 3:**
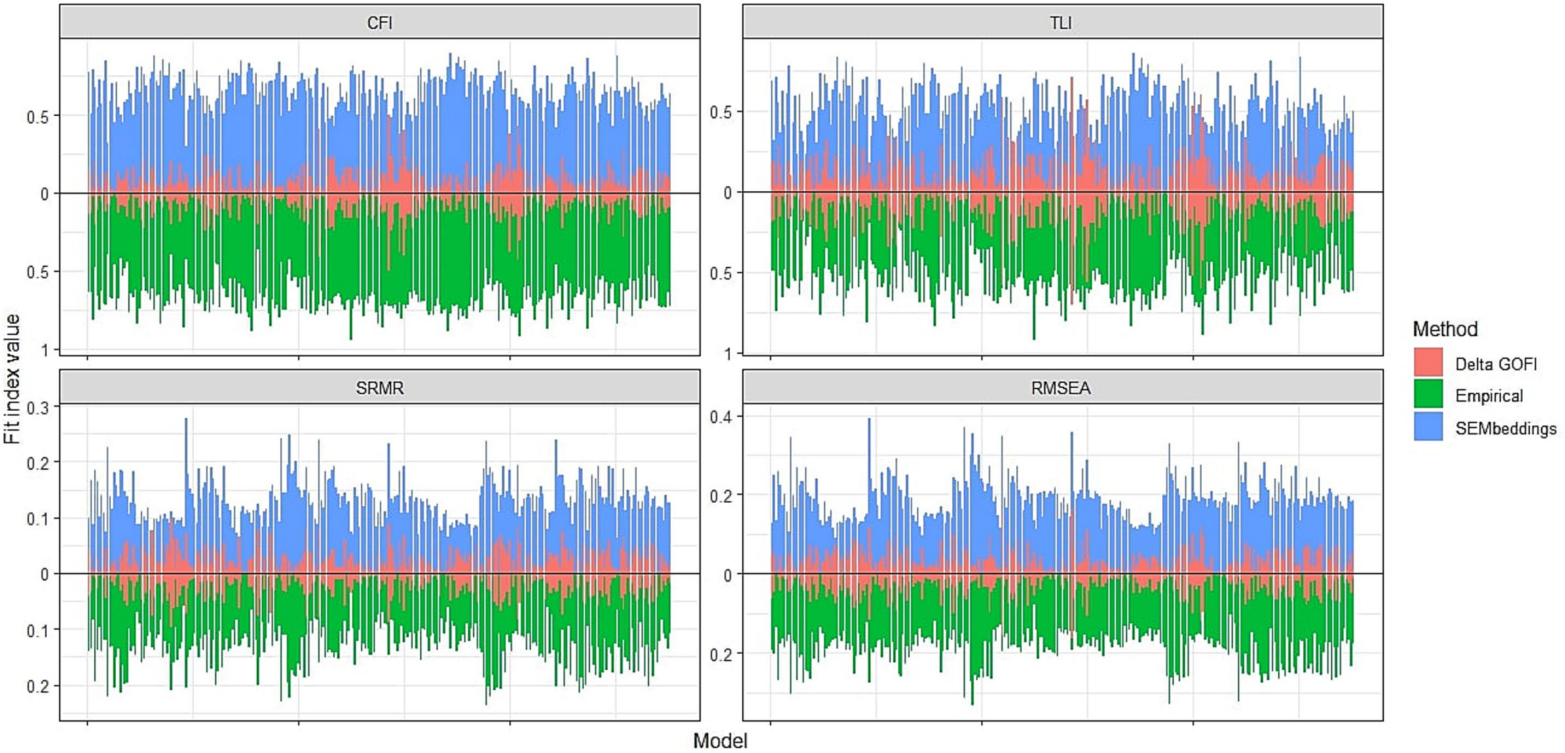
Fit indices obtained from the two methods and their absolute difference. Delta GOFI, delta goodness of fit index.

Although these results are encouraging, leading to correct decisions in both cases, the fit indices differed considerably between the two methods. For example, correlations between indices ranged from 0.40 to 0.61, with median differences between 0.02 and 0.10. See Table 2 and Figure 3 for a summary of these results.

**Table 2:** Summary statistics of differences and similarities between the fit indices obtained from the two methods.

	CFI	TLI	SRMR	RMSEA
Median value and standard deviation - SEMbeddings	0.66 (0.12)	0.52 (0.16)	0.13 (0.04)	0.19 (0.05)
Median value and standard deviation - Empirical	0.69 (0.10)	0.56 (0.14)	0.13 (0.04)	0.18 (0.04)
Correlation between fit indices values in the two conditions	0.40	0.40	0.61	0.58
Median absolute difference and standard deviation	0.07 (0.12)	0.10 (0.17)	0.02 (0.03)	0.03 (0.04)
Maximum absolute difference	0.50	0.71	0.10	0.16

#### 3.2.2 Unidimensional models

To further evaluate the performance of SEMbeddings, we assessed the unidimensionality of each individual scale. For this purpose, we fitted 24 separate Confirmatory Factor Analyses (CFAs), one for each strength, using the cosine similarity matrix as the starting covariance matrix. In this case, each model represents the theoretically correct model and should fit the data well if the model is correct. The results of these 24 models are summarized in Table 3.

**Table 3:** Fit indices of the 24 unidimensional models and their difference in the two methods (Δ).

Strength	CFI	TLI	SRMR	RMSEA
Appreciation of beauty and excellence	0.99; 0.99	0.96; 0.98	0.02; 0.02	0.08; 0.06
	Δ = −0.01	Δ = −0.02	Δ = 0	Δ = 0.02
Bravery	0.99; 1.00	0.96; 1.00	0.02; 0.01	0.05; 0.02
	Δ = −0.01	Δ = −0.04	Δ = 0.01	Δ = 0.03
Creativity	0.93; 0.96	0.78; 0.87	0.06; 0.04	0.23; 0.15
	Δ = −0.03	Δ = −0.09	Δ = 0.02	Δ = 0.07
Curiosity	0.95; 1.00	0.86; 1.00	0.03; 0.01	0.10; 0.01
	Δ = −0.05	Δ = −0.14	Δ = 0.03	Δ = 0.09
Fairness	0.90; 0.93	0.70; 0.78	0.08; 0.05	0.39; 0.24
	Δ = −0.03	Δ = −0.08	Δ = 0.03	Δ = 0.14
Forgiveness	1.00; 0.99	1.00; 0.98	0.01; 0.01	0.02; 0.05
	Δ = 0.01	Δ = 0.02	Δ = −0.01	Δ = −0.03
Gratitude	0.92; 1.00	0.76; 0.99	0.05; 0.01	0.18; 0.04
	Δ = −0.08	Δ = −0.23	Δ = 0.04	Δ = 0.15
Honesty	0.84; 0.94	0.51; 0.81	0.07; 0.05	0.25; 0.15
	Δ = −0.1	Δ = −0.29	Δ = 0.02	Δ = 0.1
Hope	0.91; 0.98	0.72; 0.93	0.05; 0.03	0.16; 0.10
	Δ = −0.07	Δ = −0.21	Δ = 0.03	Δ = 0.06
Humility	1.00; 0.99	0.99; 0.98	0.01; 0.01	0.02; 0.04
	Δ = 0	Δ = 0.01	Δ = 0	Δ = −0.02
Humor	0.99; 0.99	0.96; 0.98	0.04; 0.02	0.12; 0.08
	Δ = 0	Δ = −0.01	Δ = 0.02	Δ = 0.03
Judgment	0.99; 0.98	0.97; 0.95	0.02; 0.02	0.06; 0.07
	Δ = 0.01	Δ = 0.03	Δ = 0	Δ = −0.02
Kindness	0.94; 0.99	0.83; 0.96	0.04; 0.02	0.13; 0.07
	Δ = −0.04	Δ = −0.13	Δ = 0.02	Δ = 0.06
Leadership	1.00; 0.99	1.00; 0.97	0.00; 0.02	0.01; 0.08
	Δ = 0.01	Δ = 0.03	Δ = −0.01	Δ = −0.07
Love	0.97; 1.00	0.92; 1.00	0.03; 0.01	0.17; 0.03
	Δ = −0.02	Δ = −0.07	Δ = 0.02	Δ = 0.14
Love of Learning	0.96; 0.99	0.89; 0.96	0.04; 0.02	0.15; 0.09
	Δ = −0.02	Δ = −0.07	Δ = 0.02	Δ = 0.06
Perseverance	0.97; 1.00	0.92; 0.99	0.03; 0.01	0.12; 0.05
	Δ = −0.02	Δ = −0.07	Δ = 0.02	Δ = 0.07
Perspective	0.99; 1.00	0.96; 0.99	0.03; 0.01	0.10; 0.04
	Δ = −0.01	Δ = −0.03	Δ = 0.01	Δ = 0.06
Prudence	0.81; 0.97	0.43; 0.92	0.10; 0.03	0.29; 0.13
	Δ = −0.16	Δ = −0.49	Δ = 0.07	Δ = 0.16
Self-regulation	0.89; 0.93	0.68; 0.80	0.08; 0.07	0.20; 0.22
	Δ = −0.04	Δ = −0.12	Δ = 0.01	Δ = −0.02
Social intelligence	0.95; 0.99	0.85; 0.96	0.04; 0.02	0.12; 0.07
	Δ = −0.03	Δ = −0.1	Δ = 0.01	Δ = 0.05
Spirituality	0.98; 0.99	0.94; 0.96	0.03; 0.02	0.08; 0.10
	Δ = −0.01	Δ = −0.02	Δ = 0.01	Δ = −0.01
Teamwork	0.96; 0.91	0.88; 0.74	0.05; 0.05	0.15; 0.17
	Δ = 0.05	Δ = 0.14	Δ = −0.01	Δ = −0.01
Zest	0.86; 0.91	0.57; 0.72	0.08; 0.08	0.22; 0.26
	Δ = −0.05	Δ = −0.15	Δ = 0	Δ = −0.04

SEMbedding indices are reported on the left.

The analysis reveals that 11 models adequately fit the cosine similarity matrix, while 13 models exhibited at least two poor fit indices, with TLI and RMSEA consistently showing poor performance in these cases. These findings may signal potential misfit in the actual data, indicating areas where further investigation or model refinement may be warranted.

When comparing the results of the same models fitted on the empirical covariance matrix, we observe that fit indices correlate to some extent (*r* < 0.61). However, SEMbeddings tend to penalize fit indices, resulting in generally lower CFI and TLI values and generally higher SRMR and RMSEA values (see Table 3). The penalization is more pronounced for TLI (mean Δ = −0.09) compared to CFI (mean Δ = −0.03) and for RMSEA (mean Δ = 0.04) compared to SRMR (mean Δ = 0.02).

Descriptively, our analysis shows that when the model fits the SEMbeddings’ covariance matrix (11 times), it consistently fits the empirical covariance matrix, indicating no false positives. In the remaining 13 cases, 6 empirical models showed misfit, 5 empirical models exhibited perfect fit (i.e., curiosity, gratitude, kindness, love of learning, and social intelligence), and 2 empirical models showed acceptable but borderline fit (i.e., hope and prudence). Interestingly, in these divergent models, the RMSEA was generally high or slightly acceptable in the empirical model, suggesting potential unexpected correlations between the residuals of the items, even in the well-fitting models fitted on the empirical correlation matrix.

#### 3.2.3 Modification indices

Although the fit indices of SEMbeddings and empirical models do not always converge, we can gain insights into which items might be contributing to misfit using and interpreting modification indices.

##### 3.2.3.1 Models with bad fit indices in both conditions

For these models, we compared the results of SEMbeddings and empirical modification indices to see if they point to the same issues.

- Creativity: Modification indices suggest the presence of two pairs of correlated residuals: items 9 (“I am always coming up with new ways to do things”) and 11 (“My friends say that I have lots of new and different ideas”), as well as items 10 (“I pride myself on being original”) and 12 (“I am an original thinker”). The first two items may pertain to generating new ideas, while the latter two may relate to being original and distinct from others, potentially indicating a two-factor model. These findings are confirmed in the empirical model.
- Fairness: Modification indices indicate two pairs of items: items 17 (“I always treat people fairly whether I like them or not”) and 18 (“Even if I do not like someone, I treat him or her fairly”), as well as items 19 (“I treat all people equally regardless of who they might be”) and 20 (“I treat everyone the same”). The first pair may relate to treating people you do not like fairly, while the second pair pertains to treating all people equally. These results are confirmed in the empirical model.
- Honesty: In this case, the modification indices provided by SEMbeddings failed to detect the empirical correlated residuals: items 29 (“I always keep my promises”) and 32 (“My promises can be trusted”), as well as items 30 (“I believe honesty is the basis for trust”) and 31 (“I tell the truth even if it means I will get in trouble”). However, SEMbeddings showed high modification indices for all pairs in this case, making interpretation of the highest indices difficult.
- Self-regulation: Again, the two methods identified different pairs of items. The empirical suggestions are challenging to interpret, correlating item 77 (“It is easy for me to stay disciplined”) and item 78 (“I am good at finishing tasks even when I want to stop”) with items 79 (“I do not give in to temptation”) and 80 (“I am good at completing tasks no matter how difficult they are”). In contrast, SEMbeddings identified stronger correlations between items 78 and 80, both of which relate to task completion, potentially providing a useful, though not strictly necessary, suggestion.
- Teamwork: Modification indices detected correlated residuals between items 89 (“It is important to me to respect decisions made by my group”) and 90 (“Without exception, I support my teammates or fellow group members”), which relate to group dynamics, and items 91 (“I work at my very best when I am a group member”) and 92 (“I really enjoy being a part of a group”), which pertain to supporting group members and decisions. These results are confirmed in the empirical model.
- Zest: Modification indices also identified excessive similarity between items 94 (“I awaken with a sense of excitement about the day’s possibilities”) and 95 (“I am genuinely excited to start each day”), both of which pertain to the excitement of starting each day, as well as items 93 (“I have lots of energy”) and 96, which relate to general activity levels. These findings are confirmed in the empirical model.

##### 3.2.3.2 Models with bad fit indices in the SEMbeddings condition only

Examining models that exhibit misfit only when using the cosine similarity matrix may still provide valuable insights for item modification, even if the model perfectly fits the data. We thus explored their modification indices.

- Curiosity: In this case, modification indices highlight a similarity between items 14 (“I have many interests”) and 16 (“I am excited by many different activities”), both of which pertain to being attracted to various activities or things. This suggestion may be useful, even though the empirical model perfectly fits the data.
- Gratitude: Here, modification indices indicate a similarity between items 26 (“I have been richly blessed in my life”) and 28 (“At least once a day, I stop and count my blessings”), both related to feeling blessed. This suggestion may also be valuable, despite the empirical model perfectly fitting the data and showing small modification indices (but in the same direction of the SEMbedding model).
- Hope: The modification indices of the hope scale calculated using SEMbeddings reveal the highest residual correlations between items 35 (“I know that I will succeed with the goals I set for myself”) and 36 (“Despite challenges, I always remain hopeful about the future”), both reflecting positive views of the future. Additionally, items 33 (“I can always find the positive in what seems negative to others”) and 34 (“If I feel down, I always think about what is good in my life”) relate to the ability to find positives in negative situations. The same results are observed in the empirical modification indices, where the RMSEA is not acceptable.
- Kindness: Modification indices in this case clearly differentiate between two sets of items: those referring to friends, namely item 49 (“I am never too busy to help a friend”) and item 52 (“I really enjoy doing small favors for friends”), and those focused on helping people in need, namely item 50 (“I go out of my way to cheer up people who appear down”) and item 51 (“I always try to help people in need”). Interestingly, the empirical model, which showed an acceptable but high RMSEA, corroborates this finding.
- Love of learning: In this instance, modification indices point to the same items as the empirical models; however, interpreting them clearly proves challenging, as all pairs of items exhibit high modification indices, making it difficult to understand the underlying issues.
- Prudence: For prudence, two pairs of items showed very high modification indices: items 73 (“I always make careful choices”) and 74 (“I am a very careful person”), as well as items 75 (“I think through the consequences every time before I act”) and 76 (“I always think before I speak”). These pairs can be distinctly categorized into (a) being careful and (b) thinking before acting. This suggestion is supported by the empirical model’s modification indices, which also yielded a particularly high RMSEA.
- Social intelligence: Modification indices here clearly separate two sets of items referring to feelings: item 83 (“I am good at sensing what other people are feeling”) and item 84 (“I always know what to say to make people feel good”), and those reflecting good social interaction skills, such as item 81 (“I always get along well with people I have just met”) and item 82 (“I have the ability to make other people feel interesting”). Notably, the empirical model, which showed an acceptable but high RMSEA, reveals the same results.

In essence, despite some exception, the modification indices of the SEMbedding models accurately identify items that may exhibit excessive residual correlations.

## 4 Discussion

Test development and validation is a resource-intensive process involving item development, data collection, and analysis. Failing to validate a questionnaire due to poor model fit requires model or questionnaire modification and new data collections. Here we propose that LLMs can be used to inspect model fit to the data *a priori* to minimize the likelihood of encountering non-fitting models. Utilizing item embeddings, we can compute the cosine similarity matrix between items and fit all necessary models before data collection solely based on this similarity matrix.

To test this assertion, we employed the 96 items of the VIA-IS-P (McGrath, 2019) and compared the results obtained from the proposed tool with those from 31,697 participants who completed the VIA-IS-P.

First of all, we confirmed that cosine similarities of item embeddings resemble empirical item correlations (Hommel and Arslan, 2024). In our case, the correlation between the two lower triangles of the matrices was 0.67. This suggests that we can predict the matrix of items correlations from the embeddings of the items, but such prediction will not be perfect, and the correlations obtained should be used with caution. The cosine similarity matrix can also be descriptively used to detect items or scales that show substantial semantic overlap or cross-loadings with other scales. For example, they might be inspected to ensure that items from a subscale demonstrate the strongest similarities with items from their intended subscale rather than with others. In our case, for example, we might note that the self-regulation items show substantial overlap with the items from the perseverance subscale.

If we accept that the cosine similarity matrix of items embeddings can mimic an empirical covariance matrix of the items, we can proceed to apply the same analysis typically conducted on empirical data to the cosine similarity matrix. We labeled this process “*SEMbedding*.”

### 4.1 Detecting wrong models with SEMbeddings

A preliminary assessment of the potential of SEMbeddings involved testing its capability to identify blatantly wrong models. We achieved this by fitting 276 incorrect confirmatory factor analysis (CFA) models using both the cosine similarity matrix and the empirical correlation matrix. Our findings demonstrated that the proposed tool is proficient in detecting large model misspecifications. Specifically, the fit indices for all 276 models consistently indicated poor fit (with the exception of the SRMR), aligning almost perfectly (> 93% of the time) with the fit indices obtained from the corresponding models fitted on the empirical correlation matrix. Therefore, we can confidently assert that SEMbeddings effectively detected blatantly wrong models. However, the fit indices obtained in the two conditions were largely different (see Table 2 and Figure 3) and showed an unsatisfactory correlation (*r* < 0.62).

Following this test of baseline performance, we advanced to a more rigorous and practical scenario: identifying correct or slightly misspecified models of the unidimensional character strengths scales.

### 4.2 SEMbeddings for unidimensional scales

Our findings indicate that employing SEMbeddings can yield valuable insights into the factorial validity of scales even before data collection. However, these results should be interpreted with caution, as false negatives can often arise. Specifically, our CFA models fitted on the cosine similarity matrix show that all models exhibiting adequate fit using this matrix also demonstrate good fit on the empirical correlation matrix. Conversely, while we would conclude that the remaining 13 models do not fit the data, only 6 of these models failed to achieve adequate fit when using the empirical correlation matrix. However, it is noteworthy that in several cases, the RMSEA of these models fitted on the empirical correlation matrix remained high, suggesting that there is room for improvement and SEMbeddings could have detected it.

In summary, while SEMbeddings provide valuable information regarding model fit to empirical data, relying solely on classical fit indices may prove problematic, as erroneous conclusions about model fit can often be drawn. Therefore, it is imperative for researchers to carefully evaluate the results. Fit indices should not be interpreted rigidly based on predefined cutoffs; instead, they should be interpreted continuously to assess whether improvements can be made, and SEMbeddings should be utilized accordingly. One possible explanation for the differences between the two methods is that SEMbeddings are primarily based on semantic similarity among items, whereas human responses are influenced by multiple factors that account for true variability in the latent constructs (e.g., genetic, environmental, and social influences). Additionally, method variance, which is not directly detectable with the current LLM models, may also contribute to discrepancies.

A useful approach to better comprehend and apply these results is through the use of modification indices.

### 4.3 Modification indices

In fact, knowing that a model does not fit well has only limited utility, especially if the empirical model will probably fit better than the SEMbedding model. To understand the reason why the model is not adequate or whether we can ameliorate our model, we advocate for the use of modification indices. These can pinpoint model misspecifications and indicate where the main issues lie or where the best improvements can be made. In our analyses, modification indices clearly reveal that poorly fitting models are characterized by pairs of items exhibiting residual correlations. Identifying these issues before data collection enables researchers to reconsider item formulation or consider removing/adding redundant items that either duplicate information or expand the breadth of the construct. The suggestions provided by SEMbeddings appear reasonable and, if implemented in advance, could have enhanced scale formulation from the outset. However, the suggestions that we can obtain from the modification indices should always be carefully addressed by the researcher who should deeply reflect on whether and what should be changed. This is particularly important because SEMbeddings could wrongly detect misspecifications but could still be useful to better inspect items formulation. In any case, the use of SEMbeddings allow the researcher to explore different set of items or different formulations before data collection, thereby reducing the risk of encountering model misfit in empirical data. Researchers are encouraged to experiment with different sets of items, as this is now feasible without the need for data collection or pilot studies, which often lack sufficient power to estimate model parameters and fit accurately.

### 4.4 How to use SEMbeddings: a step-by-step guide

To summarize the procedure adopted and its utility, we here provide a bullet point list of the steps that could be taken (script and analysis are provided on OSF)^2^:

1. Define the items of interest, possibly preparing more items than needed or alternative formulations.
2. Apply an embedding model (see Wulff and Mata (2023)) to the text of all the items.
3. Calculate cosine similarity between all the embeddings (the *mpnet-personality* model already provides estimated correlations). At this point, it might be already useful to inspect the matrix of similarity to detect unexpected similarities between items of different scales or absence/low similarity between items of a single factor. These observations could guide the following analyses.
4. Fit each specific measurement model separately using a CFA for each set of items and evaluate fit indices. The CFA should be fitted using the cosine similarity matrix. If the fit indices indicate poor fit, we could further investigate the items by examining modification indices. At this point, the researchers could opt for modifying the items or the models accordingly, if they think the suggestions are meaningful.
5. We also suggest testing different set of items for each scale to eventually select the best fitting items.

### 4.5 Limitations and future directions

While our findings suggest promising utility for the proposed tool, several limitations must be acknowledged and addressed in future studies.

Firstly, SEMbeddings results do not align perfectly with empirical results. This discrepancy arises from the fact that the cosine similarity matrix and the empirical covariance matrix are not perfectly correlated. However, ongoing advancements in LLM models and embedding tools aim to enhance comparability between cosine similarities of embeddings and empirical correlations (Hommel and Arslan, 2024). New models or updates of the *mpnet-personality* model should be adopted if they will outperform the current version of *mpnet-personality.* Additionally, different similarity metrics could be adopted instead of cosine similarities, but future studies should test their performance. In any case, however, we do not believe that the two matrices will ever be identical, and the researchers should use these tools to gain more information about their items and scales and not as substitutes of empirical data collections.

Secondly, our analyses focused solely on a single questionnaire, the VIA-IS-P, which measures 24 different constructs. While this decision was made because the questionnaire covers up to 24 scales and because there is still open debate about its fit on empirical data, it limited our examination to scales consisting of only four items. Future research should investigate whether SEMbeddings perform equally well with longer scales and different constructs.

Additionally, our analyses were conducted exclusively on English items. It is imperative to explore whether LLM models exhibit similar performance with items from different languages. Notably, if LLMs are trained on texts from specific languages, it raises the possibility of conducting cross-cultural multigroup CFAs using cosine similarity matrices obtained from different languages to assess questionnaire invariance across countries.

Finally, we adopted four predetermined fit indices. Our analysis shows that they might perform differently. Future works should better explore when and why some fit indices outperform others and extend the analysis to other fit indices.

### 4.6 Conclusion

SEMbeddings, or the analysis of model misfit based on the cosine similarity matrix of items LLM embeddings, can be used to inspect whether theoretical measurement models will fit the data with the items at hand and adjust the items accordingly. In fact, although the cosine similarity of items embeddings does not perfectly correlate with empirical items’ correlations and results of CFAs fitted on the cosine similarity matrix are not always comparable with the empirical results, they tend to be more conservative. In other words, our results show that when SEMbeddings fit well, we might be quite confident that empirical data will confirm the good fit. On the other hand, if they do not fit well, they could still provide useful information and, when combined with the use of modification indices and careful researchers’ supervision, SEMbeddings could be an additional and useful tool for researchers that are developing new questionnaires to decide whether the generated items are satisfactory or should be amended before starting data collection. Although this process does not ensure success with empirical data nor substitute it, it surely decreases chances of failure.

## Data Availability

Raw data could not be made directly available because of the privacy policies of the VIA Institute on Character. However, processed data and code are available on OSF at https://doi.org/10.17605/OSF.IO/7WEHN.

## Footnotes

^1^ https://huggingface.co/dwulff/mpnet-personality

^2^ https://osf.io/7wehn/?view_only=4736a079fb014c0ea5b93e7f484c55e6
